# Influence of the Mozzarella Type on Chemical and Sensory Properties of “Pizza Margherita”

**DOI:** 10.3390/foods13020209

**Published:** 2024-01-09

**Authors:** Amalia Piscopo, Antonio Mincione, Carmine Summo, Roccangelo Silletti, Corinne Giacondino, Ilenia Rocco, Antonella Pasqualone

**Affiliations:** 1 Department AGRARIA, University Mediterranea of Reggio Calabria, via dell’Università 25, 89124 Reggio Calabria, Italy; amalia.piscopo@unirc.it (A.P.); corinne.giacondino@unirc.it (C.G.); ileniarocco@hotmail.it (I.R.); 2 Department of Soil, Plant and Food Science (DISSPA), University of Bari Aldo Moro, via Amendola, 165/a, 70126 Bari, Italy; carmine.summo@uniba.it (C.S.); roccangelo.silletti@uniba.it (R.S.); antonella.pasqualone@uniba.it (A.P.)

**Keywords:** Mozzarella di Bufala, Fiordilatte mozzarella, Pizza mozzarella, oxidation, polar compounds, volatile compounds, α-tocopherol, sensory analysis

## Abstract

Background: According to Neapolitan Pizza Traditional Specialty Guaranteed (TSG) regulation, Mozzarella di Bufala Campana and Fiordilatte mozzarella are the exclusive cheeses to be used, together with tomato and extra virgin olive oil (EVOO), to season pizza in the “Margherita” variant. However, the so-called “Pizza mozzarella”, that is a diary product having lower moisture content and a longer shelf life than Mozzarella di Bufala Campana and Fiordilatte mozzarella, is widely used in many pizzerias, both in Italy and abroad. Therefore, we investigated its quality, in comparison with Mozzarella di Bufala and Fiordilatte mozzarella, as well as its effect on the quality of the Margherita pizza. Methods: Chemical and sensory analyses were conducted on mozzarella samples and on baked pizza topping samples. Results: The results revealed a better quality of pizza with Mozzarella di Bufala and Fiordilatte mozzarella for their higher antioxidant activity, oxidative stability and lower amount of undesired volatile compounds. Conclusions: The use of Mozzarella di Bufala and Fiordilatte mozzarella in the preparation of Margherita pizza improves its quality, especially if these mozzarella types are combined with other high-quality ingredients, namely tomato sauce and EVOO, characterized by the presence of antioxidant compounds (e.g., α-tocopherol not affected by the heat treatment of pizza baking.

## 1. Introduction

Neapolitan pizza is one of the most popular products of Italian culinary landscape and is widespread all over the world. What we know today is the evolution of a food with ancient origins that has its roots in the past. The official invention of pizza, much appreciated by the then sovereign of Italy, Queen Margherita di Savoia, to whom the pizza maker dedicated his preparation, seems to date back to June 1889. “Pizza Margherita” was seasoned with tomato, mozzarella and a few basil leaves, deliberately recalling the colors of the Italian flag [1]. Pizza Margherita is one of the two official variants of Neapolitan pizza, with the other named “Pizza marinara”. These two variants are differently seasoned but their main characteristics are the same: rounded shape with a maximum diameter of 35 cm, having a central part approximately 0.25 cm thick, evenly covered by the topping, and with a raised edge (in Italian “*cornicione*”) 1–2 cm large, regularly honeycombed, golden in color and free of bubbles and burns [1]. Neapolitan pizza is cooked exclusively in wood-burning ovens for a maximum time of 60–90 s and has peculiar sensory features. It must be soft, fragrant and easily foldable. Its characteristic flavor derives from the balanced combination of the flavor of the crust (which must have the typical flavor of well-leavened and well-baked bread), with the sour note of the tomato, the spicy note of oregano, garlic or basil and finally the cheesy flavor of cooked mozzarella [1].

Mozzarella is one of its main ingredients which, during baking, melts and becomes a perfect base for binding the other ingredients together and with the crust. Choosing the right mozzarella type is therefore essential for preparing an excellent pizza.

The name “mozzarella” derives from the Italian verb “*mozzare*” (meaning to cut) and refers to an operation that is carried out during the production of this kind of cheese, consisting of cutting a piece of the stretched curd with the hands, and then shaping it manually to obtain mozzarella [2]. Commercially available types of mozzarella are classified according to their moisture level and type of milk used. They are Fiordilatte mozzarella, from cow’s milk, and Mozzarella di Bufala, from buffalo’s milk, both with a moisture content of 52% or more, also named “fresh mozzarella” owing to their short shelf life. Pizza makers, however, prefer using low-moisture mozzarella, named “Pizza mozzarella”, with a moisture content between 42% and 45% and longer shelf life [3].

Mozzarella di Bufala Campana is an excellence in the field of Italian dairy products, recognized with Protected Designation of Origin (PDO) by the European Union (EU), derived from buffalos raised in a specific and restricted area in the Campania region and in some geographical areas of the Lazio, Molise and Puglia regions, according to the European Economic Community (EEC) Regulation No. 1107/96. Buffalo milk is richer in proteins, lipids and calcium than cow’s milk, but does not contain β-carotene [4]. In addition to the delimitation of the production area, milk composition is rigorously ruled, i.e., minimum levels of fats and proteins are set, as well as the maximum period of time for use after milking. Also, the general procedure is carefully ruled, e.g., the temperature range for adding the animal rennet, etc. [5]. Mozzarella di Bufala Campana must be made with fresh and whole buffalo milk only. The processing involves the use of raw milk, possibly thermized or pasteurized. The final product is white, creamed, melted and sweet [6].

Fiordilatte mozzarella, instead, is a stretched curd cheese made from cow’s milk. The texture is soft, elastic, without holes and white in color with possible pale yellow hues. It is produced from pasteurized milk [7].

Mozzarella for pizza, commonly named Pizza mozzarella, has a lower moisture content and a longer shelf life than Mozzarella di Bufala Campana or Fiordilatte mozzarella. It is a cheaper product, usually not intended for direct consumption but mostly for dressing pizza, marketed already cut as “*julienne*” (ready for use) or entire, as a cylindrical loaf, while Mozzarella di Bufala Campana and Fiordilatte mozzarella are typically spherical. The Pizza mozzarella is prepared with partially skimmed milk, according to the degree of fat desired in the finished product. The curd, partially broken and cut into slices, is left to drain longer than the softer Mozzarella di Bufala Campana and Fiordilatte mozzarella, and the curd, after stretching, is salted. Pizza mozzarella has a closed and uniform structure which is easy to slice. For optimal use in pizzerias, once packaged it must age for at least 15 days at temperatures between 4 and 10 °C. The ever-increasing interest by the food industry in this type of mozzarella has led to the development of numerous innovations which, although being specific for Pizza mozzarella, can actually also be applied to other types of stretched curd cheeses [8].

In recent years, increased interest has been shown in healthy food capable of conferring, in addition to the basic nutritional functions, further physiological and biochemical benefits, such as the prevention and/or treatment of a vast range of pathologies. For this reason, more and more attention is paid to the quality of the raw materials and to the choice of preparation and baking methods able to preserve their original characteristics. The baking process generally affects the antioxidant compounds, which are important for both nutritional and functional purposes and positively influence the oxidative stability and shelf life of food products.

Lipid oxidation is, in fact, the main cause of the chemical deterioration of foods, including dairy products, which negatively affects the nutritional value and sensory properties. The occurrence of this phenomenon depends on the fatty acid composition, the presence of metal ions and the concentration of antioxidant compounds. The oxidative stability of milk and its derivatives derives from the balance among antioxidant and pro-oxidant factors. Among the compounds implicated in antioxidant processes are vitamin A and vitamin E. These are fat-soluble molecules capable of preventing oxidative damage caused by free radicals, the latter being highly unstable, and reactive chemical species capable of significantly contributing to the pathogenesis of numerous chronic and degenerative dysfunctions, such as inflammation, atherosclerosis, cardiac damage, ischemia, diabetes mellitus and cancer [9]. In particular, α-tocopherol can be considered one of the most important and powerful fat-soluble antioxidants present in the membrane of milk fat globules.

A few studies have focused on the nutraceutical potential of pizza. Often, in fact, the antioxidant composition is determined on the individual ingredients and the transformations produced by baking techniques are not considered. Some recent papers have evaluated the effect of the type of oil used on the physico-chemical and nutritional composition of pizza [10,11]. However, to the best of our knowledge, no studies on the influence of the type of mozzarella on the qualitative characteristics of pizza are currently published. The aim of this work is, therefore, to enrich knowledge on the chemical composition and sensory profiles of Pizza Margherita prepared with different cheese, namely Mozzarella di Bufala, Fiordilatte mozzarella and Pizza mozzarella. Particular attention was paid to studying the effect of baking on the characteristics of the topping.

## 2. Materials and Methods

### 2.1. Sample Preparation 

**Figure 1:**
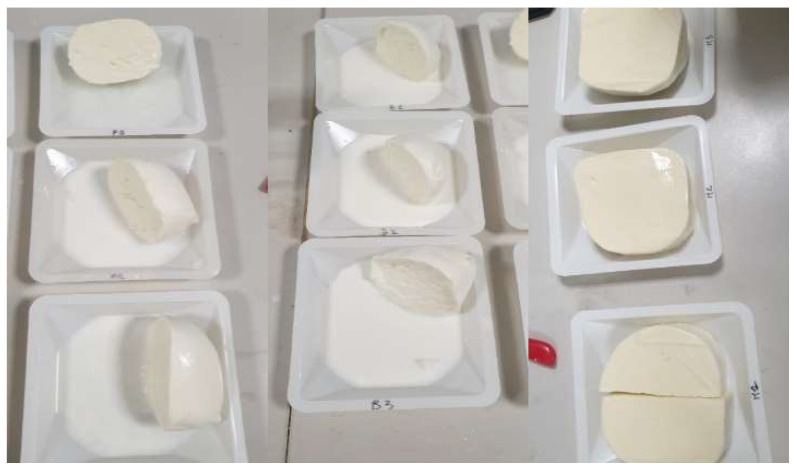
Mozzarella samples used in the experimental trials. From left to right: Fiordilatte mozzarella, Mozzarella di Bufala and Pizza mozzarella.

The production of pizza samples was carried out in a traditional pizzeria located in Reggio Calabria (Italy) using raw materials and following methods prescribed by the Production Specifications for Neapolitan Pizza TSG [1]: 1.7/1.8 kg of flour was used for 1 L of water. After preparing the dough, leavening and rolling out, the pizza crust was seasoned with tomato sauce, 4–5 g of extra virgin olive oil and mozzarella. Three types of pizza were prepared, each with a specific type of mozzarella: Fiordilatte mozzarella, Mozzarella di Bufala and Pizza mozzarella (all from Delizie della Natura, Reggio Calabria, Italy) (Figure 1). After baking, the pizza samples were packaged in cardboard boxes to be delivered to the Food Technologies Laboratory of the “Mediterranea” University of Reggio Calabria (Reggio Calabria, Italy) for the qualitative analyses. 

### 2.2. Analyses of Pizza Topping Ingredients and Topping Mix

Each ingredient of the topping, uncooked, was analyzed separately: mozzarella; tomato sauce; extra virgin olive oil. Then, the topping mix composed of mozzarella + tomato sauce + olive oil was scratched from pizza samples after baking and was analyzed to compare the physical-chemical characteristics of the ingredients before and after baking.

Below are described the analyses that were performed both on the individual ingredients and on the topping mix.

#### 2.2.1. Physico-Chemical Analyses

The determination of the colorimetric parameters of mozzarella samples was carried out using a Konica Minolta CM-700d (Tokyo, Japan) colorimeter in the CIELab color space [12]. For each mozzarella sample, five color readings were taken at different points on the external surface. Subsequently the mozzarella was cut in half to assess the internal color with the same procedure carried out for the external surface. 

An aliquot of sample (10 g of mozzarella and 5 g of tomato sauce and pizza seasoning) was subjected to dehydration at a temperature of 105 °C in an oven until reaching the constant weight for the determination of the dry matter (d.m.) and moisture content. Aqueous mozzarella extract was obtained combining 10 g of the sample with 20 mL distilled water, centrifuging at 5000 rpm at room temperature, filtering by filter paper (0.45 μm) and bringing to 50 mL volume with distilled water. The analysis of titratable acidity (% lactic acid) and pH were conducted according to the Association of Official Agricultural Chemists (AOAC) methods [13,14]. 

The lipid fraction was extracted following the Folch method [15] and enabled the extraction of the organic fraction from the mozzarella and topping mix samples, which was expressed as % of lipids.

The previously extracted lipid fractions were subjected to analysis of peroxide value (PV) according to Regulation (EC) No. 61/2011 on oils and fats [16]. The results were expressed as mEq of oxygen per kg (mEq O_2_/kg). The determination of the p-anisidine value (p-AV) was carried out as described by Steele [17]. 

The oxidative stability of mozzarella and topping mix samples was evaluated using the Oxitest instrument (Oxidation Test Reactor, Velp Scientifica, Milan, Italy) with the use of the specific software OXISoftTM, version 3.0.0. In total, 10 g of sample was used, arranged uniformly in the appropriate thermostated chambers, and the conditions suitable for the test were subsequently set, which included an overpressure of pure oxygen at 6 bar, degree 5.0 and a constant temperature of 90 °C. The results are expressed through the induction period (IP), i.e., the time necessary to start oxidation and which corresponds to a level of rancidity that is detected through a sudden change in the oxidation rate. The oxidative stability of the sample is proportional to the induction period.

The polar compounds were analyzed on pizza topping samples previously extracted as reported in [18].

#### 2.2.2. Analysis of α-Tocopherol and Antioxidant Activity

The concentration of α-tocopherol was quantified as reported by Bakre et al. [19], with appropriate modifications. An aliquot of sample (1 mL for olive oil and for the lipid fraction of mozzarella and topping samples; 500 µL for the tomato sauce) was diluted with isopropanol, homogenized and filtered through regenerated cellulose filters (pore size of 0.45 μm). A 5 μL aliquot of sample was injected into a high-pressure liquid chromatography system (UHPLC PLATINblue, Knauer, Germany) which uses a Knauer C18 column (1.8 μm, 100 × 2 mm) and a detector fluorescence model RF-20A/RF-20Axs. The flow rate was 0.5 mL min^−1^, and the mobile phase was composed of a mixture of methanol/acetonitrile (1:1). The detector was set at 295 nm in excitation and 325 nm in emission. The identification and quantification analysis of the compound was conducted by comparing the retention times with reference to the calibration line constructed using pure α-tocopherol as a standard (1–200 mg kg^−1^). The results were expressed as mg of α-tocopherol per kg of sample. 

The antioxidant activity of the samples was determined after extraction, as follows. Five g of sample (mozzarella or topping) were extracted with 25 mL of methanol (80:20 *v*:*v*), homogenized with ultraturrax and centrifuged at 9000 rpm for 10 min. The supernatant was then filtered on regenerated cellulose-based filters (0.45 μm pore size) and used to determine the antioxidant activity. The DPPH and ABTS assays were conducted according to the methods reported by Brand-Williams et al. [20] and Re et al., appropriately modified [21]. The results were expressed as Trolox equivalents (µM TE g^−1^).

#### 2.2.3. Determination of Volatile Compounds 

The volatile compounds of ingredients and toppings were analyzed following the method reported by [22]. 

### 2.3. Sensory Analysis of Pizza Samples

Descriptive sensory analysis of Pizza Margherita samples was performed with a panel composed of 12 trained panelists (3 males, 9 females, 22 to 42 years old, recruited among students and staff of the “Mediterranea” University of Reggio Calabria, Italy). Pizza samples prepared with each of the three different mozzarella types were served in a dedicated and isolated area of the producing pizzeria randomly in a single session, immediately after baking. Panelists rated pizza samples on a 10-point structured scale for appearance, olfactory, taste and textural descriptors. A preliminary training tasting session on pizza sensory attributes was made one day before the actual test. During this session, descriptors obtained from [11] were integrated with new topping descriptors, shown in Table 1. Taste and texture were additionally evaluated on the topping alone. The minimum score (0) indicated the absence of the attribute, while the maximum score (9) indicated a very intense attribute. 

Panelists cleansed their tongue with mineral sparkling water among samples. Mean results were plotted as spider plots.

### 2.4. Statistical Analysis

The results were expressed as the mean ± standard deviation of three replicates. The data were submitted to a one-way analysis of variance (ANOVA) in order to highlight any significant differences. Tukey’s test was applied for multiple comparisons through Minitab version 16.0 statistical software (Minitab Inc., State College, PA, USA).

## 3. Results and Discussion

### 3.1. Analyses of Mozzarella Samples

The results of the colorimetric analyses on mozzarella samples are reported in Table 2. Very similar results of Lightness (L*) in the outer and inner layers of Fiordilatte and Mozzarella di Bufala were observed, whereas lower values were detected with Pizza mozzarella samples. For Fiordilatte and Mozzarella di Bufala, the a* values were slightly negative (about −0.2 and −0.9, respectively), with no difference between the surface and the inside. In Pizza mozzarella, however, the values of the a* component, both internal and external, were positive (0.5 and 0.9). However, these minimal differences could not be detected by eye but only instrumentally. 

Finally, the b* values of Fiordilatte and Mozzarella di Bufala were very similar to each other and all positive (range 6–8). At the same time, there was a difference, not too marked, between the external and the internal part, with the former showing slightly lower b* values than the latter because the interior of both types of mozzarella had a higher yellow component than the outside. In Pizza mozzarella, instead, opposite results were observed, with the outer part slightly more yellow than the inside. Finally, the analysis of color saturation (Chroma) confirmed the color similarity between Fiordilatte and Mozzarella di Bufala with respect to Pizza mozzarella. 

Also, the results of the chemical analyses evidenced the significant differences between Pizza mozzarella, Fiordilatte mozzarella and Mozzarella di Bufala (Table 3). As expected, Pizza mozzarella was characterized by a significantly lower moisture content than the other two types of mozzarella. The moisture content of Mozzarella di Bufala fell within the parameters established by the PDO specification (moisture content ≤ 65%) [23]. As regards Fiordilatte mozzarella, it should have a moisture content ≤ 60%, as was observed in the sample used in the experiments [24]. 

Consistently with a higher fat content present in the original milk, Mozzarella di Bufala stood out from other samples for this nutrient [25], while the lowest fat content was found in Pizza mozzarella, a parameter typically kept low during its production.

The pH values of mozzarella samples were between 5.56 and 5.91, which were in agreement with the processing technology of every type of mozzarella, which involves a fermentation phase of the curd prior to stretching. The value of free acidity of the three types of mozzarella varied from 0.17 to 0.49, expressed as lactic acid %. Produced during lactic fermentation carried out by the acidogenic bacteria of the *Lactobacillus* and *Streptococcus* genus, lactic acid is partially lost by solubilization during the stretching phase and immersion in hot water at 90 °C. Mozzarella samples significantly differed for this parameter, with a higher titratable acidity in Pizza mozzarella and a lower value in Fiordilatte mozzarella, in accordance with the literature [26].

Food products with a high fat content and obtained with certain favorable processing or storage conditions undergo the lipid oxidation process, with a deteriorating effect on their quality. In fact, oxidative events influence taste, flavor and nutritional value [22]. The peroxide number, also called peroxide value, is a parameter that indicates the degree of primary oxidation of lipids, in particular, triglycerides. The alteration of these compounds, if not controlled and limited, can lead to serious defects in the food, with worsening of the organoleptic characteristics due to the development of a perceivable rancid odor. The results observed in the mozzarella samples did not evidence significant differences, with very low peroxide values. By determining the value of *p*-anisidine, instead, it is possible to evaluate the secondary oxidation, as this index spectrophotometrically measures the products derived from the reaction between aldehydes (secondary products of the oxidative process) and *p*-anisidine. Aldehydes represent almost 50% of the volatile compounds produced during lipid oxidation [27]. Also for this parameter, the resulting values were low (1.34–1.78). Overall, both the oxidation indexes were well below the acceptability threshold [28,29].

The analysis carried out using the Oxitest made it possible to evaluate the stability to oxidation of the three types of mozzarella, in conditions of accelerated oxidation, to further quantify the effect of different mozzarella types on pizza quality. Pizza mozzarella and Fiordilatte mozzarella showed a longer induction period than Mozzarella di Bufala. The greater stability of Pizza mozzarella was probably due to its lower fat content. The Oxitest, indeed, subjects the sample to high O_2_ pressure and temperature and then monitors the sample oxidation by measuring the drop in O_2_ pressure. The O_2_ consumption of the sample, generally slower (i.e., longer induction times) in the presence of a high content of antioxidants (which was not the case of Pizza mozzarella), also depends on the lipid content of the sample; the lower the lipids, the longer the time needed to consume the O_2_. Another explanation for the oxidation stability of Pizza mozzarella could be its lower water content. According to Karel [30], high values of water activity determine an acceleration of the lipid oxidation process since water makes cell membranes more permeable, favoring the mobilization of reagents and catalysts.

On the other hand, food antioxidants protect lipids from the oxidative stress induced by processing and storage. The most represented lipophilic antioxidant in milk, involved in scavenging the peroxyl radicals, is α-tocopherol [31]. This compound was detected at levels from 2 to 3 mg/kg in Fiordilatte and Mozzarella di Bufala, while significantly lower amounts were found in Pizza mozzarella. The results were in accordance with the scientific literature [32]. The observed differences could be imputable to many factors, from the animal diet to the processing technology, with the latter related to possible oxidations.

The antioxidant activity assay against the DPPH radical did not evidence significant differences among mozzarella samples (*p* > 0.05). Values between 0.14 and 0.16 µM TE/g were observed, indicating a low reactivity with this assay, which mostly measures the antioxidants of the lipophilic matrices. The results of the ABTS assay, which is better suited to testing both hydrophilic and lipophilic substances, were significantly different (*p* < 0.01), with higher values in Pizza mozzarella (1.51 ± 0.07 µM TE/g), followed by Mozzarella di Bufala (0.71 ± 0.03 µM TE/g) and Fiordilatte mozzarella (0.68 ± 0.05 µM TE/g). These results are consistent with previous studies on lacto-fermented mozzarella [33]. The antioxidant capacity of milk and its derivatives is due to sulfur-containing amino acids (cysteine), vitamins A and E, carotenoids and enzymatic systems [34]. Vitamin A and Vitamin E are fat-soluble compounds that inhibit the chain reaction of lipid peroxidation through the elimination of various free radicals that catalyze the initiation and propagation phases. Furthermore, they allow us to prevent or limit the oxidation of fatty acids by eliminating singlet oxygen and lipoperoxides [35,36]. Balestrieri et al. [32] found higher redox markers in buffalo mozzarella compared to cow mozzarella, as reported in the present study.

The analysis of the volatile component of the mozzarella used in the pizza topping highlighted significant differences among the different samples considered (Table 4). Fiordilatte mozzarella showed higher levels of volatile compounds than Mozzarella di Bufala, including higher amounts of 3-hydroxy-2-butanone, or acetoin. This compound, imputable to the LAB starter, has a typical butterscotch flavor. Also, 2,3-butanedione (or diacetyl), which has an intensely buttery flavor, was more abundant in Fiordilatte than in Mozzarella di Bufala.

Acetic acid was found to be particularly abundant in Pizza mozzarella. This compound, which confers a typical sour flavor, commonly arises from the metabolism of heterolactic fermentation carried out by lactic acid bacteria and other adventitious bacteria. Other authors detected higher amounts of acetic acid in Pizza mozzarella compared to Fiordilatte mozzarella [37]. Pizza mozzarella also showed relevant amounts of ethanol, typically arising from fermentative activities.

Except for acetic acid, all the other acids are derived by the lipolysis of triglycerides. Each of them imparts a specific flavor. Butanoic acid is characterized by cheesy and buttery flavors, while hexanoic acid has a more pungent flavor, similar to Blue cheese. Octanoic acid contributes to wax, goat and rancid perceptions [38]. These acids, however, were detected in low amounts.

### 3.2. Tocopherol Content of Tomato Sauce and Extra Virgin Olive Oil

The analysis of tomato sauce and extra virgin olive oil was focused only on the α-tocopherol content due to its positive effect in preventing oxidation. Values of 10.91 ± 2.17 mg kg^−1^ and 192 ± 22 mg kg^−1^ were ascertained in tomato sauce and in extra virgin olive oil, respectively, in accordance with the current literature [39,40,41]. 

### 3.3. Analyses of Pizza Topping 

The principal qualitative analyses performed on pizza topping samples after baking are reported in Table 5. Moisture and lipid contents were higher in toppings with Mozzarella di Bufala and Fiordilatte mozzarella. These results mirrored those observed on the characteristics of the ingredients before baking. Significant differences were not observed among samples in the primary oxidation, whereas a significant variability was ascertained for the secondary one, expressed by the p-anisidine value. Higher values of p-AV, indeed, were found in the toppings containing Fiordilatte mozzarella and Pizza mozzarella than in the one with Mozzarella di Bufala. Despite this, the accelerated oxidation test—highly effective as it measures the oxidative stability of food as it is, without needing a preliminary extraction of fat—evidenced the lowest stability of the topping containing Pizza mozzarella. These findings are probably due to the factors already described previously, such as the content of antioxidant compounds, as well as their combination with other ingredients, such as extra virgin olive oil and tomato. In fact, the oxidative stability in the topping mixes with Fiordilatte mozzarella and Mozzarella di Bufala increased with respect to the single ingredients, highlighting a synergy between them [11]. The topping with Mozzarella di Bufala presented the highest content of α-tocopherol (6.99 ± 0.31 mg kg^−1^), followed by the topping with Fiordilatte mozzarella (6.02 ± 0.11 mg kg^−1^) and then by the topping with Pizza mozzarella (5.07 ± 0.46 mg kg^−1^), highlighting that the content of α-tocopherol was the most influential factor in determining the oxidative stability. These results are close to those reported in a study conducted by Chatterjee [42] on the quantification of vitamin E in various foods.

Antioxidant activity differences in pizza toppings were significant between samples (*p* < 0.01). With the ABTS assay, the highest antioxidant activity was observed in the topping containing Pizza mozzarella (1.31 ± 0.06 μM TE/g), followed by the topping with Fiordilatte mozzarella (0.89 ± 0.03 μM TE/g) and by the one with Mozzarella di Bufala (0.88 ± 0.02 μM TE/g). In the topping, unlike the mozzarella samples, the effect of tomato must also be taken into account. This ingredient, in fact, significantly influences the antioxidant profile of the topping. It has been reported that the heat treatment of tomato sauce significantly modifies, among other things, its chemical composition. Gahler et al., in fact, argued that the increase in the antioxidant activity of tomato sauce following baking could be due to an increase in phenolic compounds. The latter is due to the destruction of molecular structures, favoring the release of bound phenolics [43]. Other authors reported a total antioxidant activity increase after thermal processing of tomatoes at 88 °C [44]. In a previous study, we highlighted this phenomenon in the variant “Marinara” of the Neapolitan pizza [11]. However, another reason for the increase in hydrophilic antioxidant capacity could be the formation of Maillard products, with the latter being active antioxidant substances that arise from severe heat treatments, like baking. It is assumed that these compounds were present at a greater extent in the topping with Pizza mozzarella. 

A comparison of the results obtained by the two different assays shows that also for the topping, the highest values were measured by ABTS, highlighting a greater antioxidant activity expressed by the hydrophilic component of the samples. In the case of the DPPH test, the greater antioxidant activity detected in the toppings, compared to uncooked mozzarella alone (in the specific case of Fiordilatte and Mozzarella di Bufala), was, as observed for the ABTS assay, attributable to the synergy with other ingredients, namely tomato and extra virgin olive oil, with reference to the presence of lipophilic bioactive constituents.

### 3.4. Volatile Compounds of Pizza

Table 6 shows the volatile compounds of pizza topped with tomato sauce, olive oil and mozzarella cheeses, reproducing the typical Pizza Margherita. Several compounds were detected, derived from fermentations, from thermal reactions and from the oxidation of lipids, which are all reactions which occur during pizza processing. Significant differences were observed in the volatile profile as a function of the type of mozzarella cheese used. The amounts of alcohols and acetic acid reflected the differences observed in the three types of mozzarella and further increased in pizza due to an additional production of the same compounds during dough fermentation. In particular, ethanol and acetic acid and, consequently, also ethyl acetate, were significantly more abundant in the samples seasoned with Pizza mozzarella than in those with the other two types of mozzarella cheese.

2-Butanone-3-hydroxy, another fermentation-originated compound [45] which gives butter-like, cream-like notes to the odor profile [46], remained more abundant in the presence of Fiordilatte mozzarella. This compound increased with pizza-making, probably during dough leavening.

Irrespective of the mozzarella type, compared to the starting ingredient, the levels of hexanal, nonanal and hexanoic acid of the pizza topping were higher due to the oxidation of the mozzarella fatty fraction, further enhanced by the oxidation of flour lipids and olive oil added to season the pizza.

The volatile profile of pizza showed also many compounds related to thermal reactions, such as 2-methylpropanal, 2-methylbutanal, 3-methylbutanal, 2-furancarboxaldehyde (furfural), 5-methyl-2-furancarboxaldehyde (5-methylfurfural), methyl-pyrazine, 2-pentylfuran and 2-furanmethanol, that were absent or at very low amounts in the three considered typologies of mozzarella. These compounds derive from the Maillard reaction and are related to sweet, caramel odor sensations typical of baked goods [47,48].

### 3.5. Polar Compounds

The polar compounds of the lipid fraction of pizza topped with different types of mozzarella cheese are shown in Table 7. These compounds are classified as triacylglycerol oligopolymers (TAGP), oxidized triacylglycerols (Ox-TAG) and diacylglycerols (DAG) and are considered useful to ascertain the quality of lipids [49,50]. The oxidative and hydrolytic degradations underlying the formation of these compounds negatively influence the nutritional and sensory quality of food products [50]. Moderate levels of polar compounds have been already ascertained in several bakery products as an effect of processing [18,51].

The levels of polar compounds observed in the different pizza samples, in particular the TAGP, agreed with the volatile oxidation markers, e.g., the hexanal levels were lower in Mozzarella di Bufala than in Fiordilatte and Pizza mozzarella (with no significant differences between the latter two). Pizza mozzarella showed intermediate levels of Ox-TAG but exhibited the highest values of DAG. The overall levels observed for TAGP, Ox-TAG and DAG in the three pizza samples were consistent with those found in similar bakery products [22].

### 3.6. Sensory Analysis

**Figure 2:**
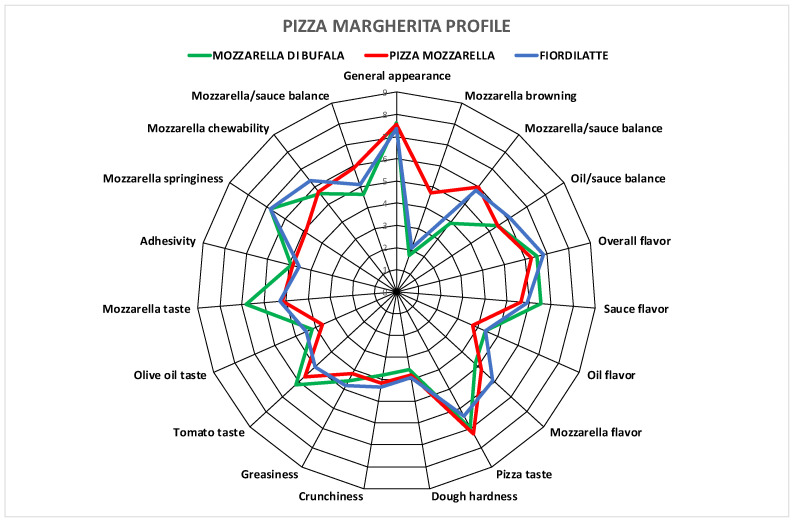
Sensory profile of Margherita-style pizza samples.

Sensory profiles for pizza samples produced with the three different types of mozzarella cheese are shown in Table 8 and Figure 2.

The main descriptors (with higher scores) were found to be general appearance, overall flavor, sauce flavor and pizza taste. Surprisingly, the results of the descriptive sensory analysis performed did not show significant differences among samples in most descriptors, with the exception of mozzarella browning, which was more intense in Pizza mozzarella, and mozzarella/tomato sauce balance descriptor, which was lower in Mozzarella di Bufala.

The mozzarella taste descriptor was scored as more intense in pizza samples prepared with Mozzarella di Bufala, but without enough statistical significance. One textural pizza descriptor, mozzarella springiness, showed lower results for the Pizza mozzarella topping, but again without enough statistical significance.

This outcome shows that the effect of different mozzarella typologies was smoothed after mixing with the other ingredients and baking; although, especially Mozzarella di Bufala showed a greater tendency to influence the visual, textural and taste aspects. Other organoleptic aspects of the product, on the other hand, were not significantly influenced.

## 4. Conclusions

The results of this scientific work show that the Margherita-style Neapolitan pizza topped with Mozzarella di Bufala and Fiordilatte mozzarella presents a superior quality in terms of the investigated chemical characteristics, in particular for higher α-tocopherol content and lower amounts of some undesirable volatile compounds. Mozzarella di Bufala also showed a greater tendency to influence the visual, textural and taste aspects, but the influence of different mozzarella cheese on the sensory features of pizza was smoothed after mixing with the other ingredients and baking.

In addition, the obtained results show that a bakery product such as Pizza Margherita has interesting nutritional and functional properties when made with high-quality ingredients which not only characterize the product for its sensory features but, in synergy with each other, maintain high this quality even after baking.

Further research could be conducted to evaluate the effect of the cheese samples on some other compositional characteristics in pizza, such as protein and ash.

## Figures and Tables

**Table 1:** List of the sensory descriptors analyzed in pizza samples.

Category	Descriptor	Definition
Appearance	General appearance	Overall appearance of pizza
	Mozzarella browning	Presence and intensity of mozzarella browning
	Mozzarella/tomato sauce balance	Visual balance between the amount of mozzarella and tomato sauce on pizza
	Oil/tomato sauce balance	Visual balance between the amount of oil and tomato sauce on pizza
Olfactory	Overall flavor	Product flavor intensity
	Sauce flavor	Intensity of sauce flavor
	Oil flavor	Intensity of oil flavor
	Mozzarella flavor	Intensity of mozzarella flavor
Taste/Pizza texture	Pizza taste	Product typical taste intensity
	Dough hardness	Overall hardness of cooked base
	Crunchiness	Teeth cutting resistance intensity
	Greasiness	Mouthfeel greasiness intensity
Taste/Topping texture	Tomato taste	Typical tomato taste intensity
	Olive oil taste	Typical olive oil taste intensity
	Mozzarella taste	Typical mozzarella taste intensity
	Adhesivity	Mouth adhesion feeling rate
	Mozzarella springiness	Mozzarella springiness feeling intensity
	Mozzarella chewability	Mozzarella chewing resistance rate
	Mozzarella/tomato sauce balance	Taste balance between mozzarella and tomato sauce

**Table 2:** Results of colorimetric analysis of the surface and interior of different types of mozzarella used in pizza seasoning.

Sample	Portion Examined							
	Outer				Inner			
	L*	a*	b*	Chroma	L*	a*	b*	Chroma
Fiordilatte mozzarella	94.33 ± 0.93 a	−0.19 ± 0.13 b	6.02 ± 2.18 b	6.02 ± 2.17 b	93.18 ± 1.00 a	−0.16 ± 0.12 b	7.66 ± 1.50 a	7.66 ± 1.50 a
Mozzarella di Bufala	94.34 ± 0.93 a	−0.93 ± 0.20 c	6.04 ± 0.57 b	6.11 ± 0.91 b	94.09 ± 1.21 a	−0.96 ± 0.14 c	7.45 ± 0.72 a	7.51 ± 0.72 a
Pizza mozzarella	88.38 ± 1.38 b	0.49 ± 0.12 a	7.82 ± 0.91 a	7.84 ± 0.58 a	89.55 ± 1.14 b	0.93 ± 0.18 a	5.36 ± 1.42 b	5.46 ± 1.36 b
Significance	**	**	**	**	**	**	**	**

Different letters in the same row correspond to significant differences. ** Significance at *p* < 0.01.

**Table 3:** Results of the chemical analyses of different types of mozzarella used in pizza seasoning.

Parameter	Fiordilatte Mozzarella	Mozzarella di Bufala	Pizza Mozzarella	Significance
Moisture (%)	59.56 ± 0.07 a	59.10 ± 2.15°	52.82 ± 0.25 b	**
Lipids (%/d.m.)	53.61 ± 0.28 b	64.13 ± 0.32°	43.20 ± 0.15 c	**
pH	5.72 ± 0.04 ab	5.56 ± 0.08b	5.91 ± 0.02 a	*
FA (lactic acid %)	0.17 ± 0.05 c	0.30 ± 0.03b	0.49 ± 0.00 a	**
PV (mEq O_2_/kg)	0.62 ± 0.18	0.49 ± 0.00	0.75 ± 0.00	n.s.
*p*-AV	1.78 ± 0.03 a	1.73 ± 0.01°	1.34 ± 0.05 b	**
Oxidative stability (h)	17.01 ± 0.01 a	11.15 ± 0.21 b	15.45 ± 0.64 a	**
α-Tocopherol (mg kg^−1^)	2.23 ± 0.06 ab	2.97 ± 0.15 a	1.65 ± 0.33 b	*
AA-DPPH (µM TE g^−1^)	0.14 ± 0.00	0.15 ± 0.01	0.16 ± 0.01	n.s.
AA-ABTS (µM TE g^−1^)	0.68 ± 0.05 b	0.71 ± 0.03 b	1.51 ± 0.08 a	**

FA = Free Acidity; PV = Peroxide Value; *p*-AV = *p*-Anisidine Value; AA = Antioxidant activity. Different letters in the same row indicate significant differences at *p* < 0.05. ** Significance at *p* < 0.01; * Significance at *p* < 0.05; n.s. not significant.

**Table 4:** Volatile compounds of different types of mozzarella used in pizza seasoning.

Volatile Compound (µg/g)	Type of Mozzarella			
	Fiordilatte Mozzarella	Mozzarella di Bufala	Pizza Mozzarella	Sign.
Alcohols				
Ethanol	0.19 ± 0.05 b	0.15 ± 0.05 b	11.22 ± 1.58 a	**
3-Methyl-1-butanol	0.17 ± 0.01 b	0.23 ± 0.17 b	1.60 ± 0.03 a	*
1-Hexanol	0.04 ± 0.01	0.05 ± 0.01	0.08 ± 0.02	n.s.
Aldehydes				
Nonanal	0.36 ± 0.03 a	0.23 ± 0.02 b	0.42 ± 0.03 a	**
2-Furancarboxaldehyde	1.25 ± 0.15 a	0.21 ± 0.08 c	0.65 ± 0.11 b	*
Benzaldehyde	0.17 ± 0.07 c	0.71 ± 0.09 a	0.54 ± 0.05 b	**
5-Methyl-2-furancarboxaldehyde	0.04 ± 0.01 b	0.08 ± 0.01 a	0.10 ± 0.01 a	*
Ketones				
3-Hydroxy-2-butanone	6.15 ± 0.62 a	3.31 ± 0.61 b	0.27 ± 0.06 c	**
2,3-Butanedione	4.49 ± 0.44 a	1.59 ± 0.21 c	2.59 ± 0.35 b	**
2-Heptanone	0.32 ± 0.02 a	0.08 ± 0.13 b	0.08 ± 0.01 b	*
6-Methyl-5-hepten-2-one	1.48 ± 0.07 a	1.77 ± 0.17 a	0.34 ± 0.08 b	*
2-Nonanone	n.d.	0.12 ± 0.03	0.10 ± 0.02	n.s.
Carboxylic acids				
Acetic acid	1.84 ± 0.20 b	1.83 ± 0.39 b	11.08 ± 3.35 a	**
Butanoic acid	0.24 ± 0.01 b	0.08 ± 0.01 c	3.19 ± 0.28 a	**
Hexanoic acid	0.20 ± 0.03 b	0.15 ± 0.02 c	0.29 ± 0.02 a	*
Octanoic acid	0.25 ± 0.04	n.d.	n.d.	-

Different letters in the same row indicate significant differences; n.d. = not detected; ** Significance at *p* < 0.01; * Significance at *p* < 0.05; n.s. not significant.

**Table 5:** Results of the chemical analyses of Margherita-style pizza topping.

Parameter	Topping with Fiordilatte Mozzarella	Topping with Mozzarella di Bufala	Topping with Pizza Mozzarella	Significance
Moisture (%)	53.04 ± 1.15	50.02 ± 0.99	49.90 ± 2.41	n.s.
Lipids (%)	18.81 ± 0.62 b	21.19 ± 0.42 a	14.40 ± 0.56 c	**
PV (mEq O_2_/kg)	0.35 ± 0.07	0.55 ± 0.07	0.55 ± 0.08	n.s
*p*-AV	12.23 ± 3.86 a	1.28 ± 0.14 b	7.10 ± 0.75 ab	*
Oxidative stability (h)	22.95 ± 0.07 a	18.75 ± 0.35 b	5.65 ± 0.49 c	**
α-tocopherol (mg kg^−1^)	6.02 ± 0.11 ab	6.99 ± 0.31 a	5.07 ± 0.46 b	*
AA-ABTS (µM TE g^−1^)	0.89 ± 0.03 b	0.88 ± 0.02 b	1.31 ± 0.07 a	**
AA-DPPH (µM TE g^−1^)	0.19 ± 0.02 a	0.17 ± 0.02 a	0.14 ± 0.01 b	**

PV = Peroxide Value; *p*-AV = *p*-Anisidine Value; AA = Antioxidant activity. Different letters in the row indicate significant differences at *p* < 0.05. ** Significance at *p* < 0.01; * Significance at *p* < 0.05; n.s. not significant.

**Table 6:** Volatile compounds of Margherita-style pizza topping.

Volatile Compound (µg/g)	Type of Topping			
	Topping with Fiordilatte Mozzarella	Topping with Mozzarella di Bufala	Topping with Pizza Mozzarella	Significance
Alcohols				
Ethanol	28.20 ± 0.94 b	25.41 ± 3.19 b	39.25 ± 2.77 a	**
2-Methyl-1-propanol	2.53 ± 0.38 b	2.88 ± 0.23 b	4.90 ± 0.41 a	*
3-Methyl-1-butanol	16.89 ± 0.55	14.05 ± 1.21	15.84 ± 5.41a	n.s.
1-Hexanol	0.62 ± 0.05	2.70 ± 0.11	2.82 ± 0.15	n.s.
(Z)-3-Hexen-1-ol	0.19 ± 0.02 c	0.22 ± 0.03 b	0.32 ± 0.03 a	*
Aldehydes				
2-Methylbutanal	10.59 ± 0.38	10.57 ± 0.24	10.36 ± 0.20	n.s.
3-Methylbutanal	3.83 ± 0.20	4.21 ± 0.50	4.43 ± 0.48	n.s.
Hexanal	1.92 ± 0.14 a	1.16 ± 0.05 b	2.12 ± 0.09 a	*
(E)-2-Hexenal	1.69 ± 0.23	1.27 ± 0.29	1.24 ± 0.15	n.s.
(E,E)-2,4-Hexadienal	n.d.	0.05 ± 0.00 a	0.01 ± 0.01 b	**
Nonanal	0.84 ± 0.14 a	0.62 ± 0.02 b	0.70 ± 0.01 a	*
2-Furancarboxaldehyde	4.75 ± 2.35 b	6.32 ± 1.99 b	14.10 ± 1.70 a	*
Benzaldehyde	2.76 ± 0.62 a	1.61 ± 0.12 b	1.81 ± 0.12 b	*
5-Methyl-2-furancarboxaldehyde	0.52 ± 0.13 c	1.26 ± 0.22 b	2.34 ± 0.18 a	**
Ketones				
Acetone	2.07 ± 0.20 ab	1.93 ± 0.09 b	2.38 ± 0.06 a	**
3-Hydroxy-2-butanone	15.53 ± 0.93 a	7.51 ± 0.44 c	8.40 ± 0.07 b	**
6-Methyl-5-hepten-2-one	8.98 ± 2.17	6.69 ± 0.32	7.53 ± 0.49	n.s.
2-Nonanone	0.32 ± 0.19	n.d.	0.22 ± 0.07	n.s.
Carboxylic acids				
Acetic acid	15.32 ± 2.19 b	11.01 ± 1.66 b	25.43 ± 2.89 a	*
Propanoic acid	n.d.	0.09 ± 0.01 b	0.13 ± 0.01 a	**
Butanoic acid	0.26 ± 0.03 b	0.30 ± 0.02 b	0.75 ± 0.08 a	*
Pentanoic acid	n.d.	0.05 ± 0.01	n.d.	-
Hexanoic acid	0.57 ± 0.02 a	0.32 ± 0.01 c	0.49 ± 0.02 b	*
Esters				
Ethyl acetate	5.01 ± 0.74 b	4.32 ± 0.41 b	8.18 ± 0.73 a	*
3-Hexen-1-ol, acetate	0.04 ± 0.01 c	0.58 ± 0.13 b	3.75 ± 0.31 a	**
Pyrazines				
Pyrazine	0.51 ± 0.11 a	0.64 ± 0.05 a	0.30 ± 0.01 b	*
Methylpyrazine	0.75 ± 0.08 b	1.52 ± 0.33 a	1.44 ± 0.05 a	*
Ethylpyrazine	0.28 ± 0.03 b	0.32 ± 0.02 b	0.53 ± 0.01 a	*
Furans				
1-(2-furanyl)-Ethanone	0.89 ± 0.11	0.76 ± 0.13	0.94 ± 0.26	n.s.
2-Penthylfuran	0.41 ± 0.11 b	n.d.	0.83 ± 0.07 a	**
2-Furanmethanol	0.36 ± 0.32 c	1.46 ± 0.08 a	0.93 ± 0.04 b	**

n.d. not detected. Different letters within rows indicate significant differences. ** Significance at *p* < 0.01; * Significance at *p* < 0.05; n.s. not significant.

**Table 7:** Variation of the polar compounds of Margherita-style pizza topping.

Polar Compound (g/100 g)	Topping with Fiordilatte Mozzarella	Topping with Mozzarella di Bufala	Topping with Pizza Mozzarella	Significance
TAGP	0.19 ± 0.01 ab	0.12 ± 0.01 c	0.16 ± 0.01 b	*
Ox-TAG	0.71 ± 0.01 a	0.59 ± 0.02 c	0.65 ± 0.02 b	*
DAG	2.39 ± 0.04 b	2.18 ± 0.04 c	2.87 ± 0.05 a	**

TAGP = triacylglycerol oligopolymers; Ox-TAG = oxidized triacylglycerols; DAG = diacylglycerols. Different letters in the row indicate significant differences. ** Significance at *p* < 0.01; * Significance at *p* < 0.05.

**Table 8:** Results of the descriptive sensory analysis of Margherita-style pizza samples.

Descriptor	Fiordilatte Mozzarella	Mozzarelladi Bufala	Pizza Mozzarella	Significance
Appearance				
General appearance	7.58 ± 2.28	7.58 ± 1.62	7.67 ± 1.23	n.s.
Mozzarella browning	2.00 ± 1.21 a	1.67 ± 1.44 a	4.42 ± 2.91 b	*
Mozzarella/tomato sauce balance	5.75 ± 0.87 a	3.83 ± 1.59 b	5.92 ± 1.44 a	*
Oil/tomato sauce balance	6.00 ± 0.95	5.42 ± 1.51	5.42 ± 1.31	n.s.
Olfactory				
Overall flavor	7.00 ± 1.81	6.75 ± 1.87	6.42 ± 1.62	n.s.
Sauce flavor	5.92 ± 1.62	6.58 ± 1.83	5.67 ± 2.27	n.s.
Oil flavor	4.17 ± 1.90	4.25 ± 1.76	3.58 ± 1.83	n.s.
Mozzarella flavor	5.50 ± 2.39	4.67 ± 1.50	4.92 ± 2.50	n.s.
Taste/Pizza texture				
Pizza taste	6.67 ± 1.82	7.17 ± 0.94	7.50 ± 1.93	n.s.
Dough hardness	3.75 ± 1.71	3.42 ± 1.78	3.58 ± 1.56	n.s.
Crunchiness	4.17 ± 1.90	3.67 ± 1.07	4.00 ± 2.13	n.s.
Greasiness	4.75 ± 1.49	4.50 ± 2.02	4.17 ± 2.33	n.s.
Taste/Topping texture				
Tomato taste	4.92 ± 1.73	6.00 ± 1.81	5.67 ± 2.02	n.s.
Olive oil taste	4.25 ± 1.60	3.92 ± 1.56	3.50 ± 1.00	n.s.
Mozzarella taste	5.08 ± 2.15	6.83 ± 1.95	5.33 ± 2.10	n.s.
Adhesivity	4.25 ± 2.22	4.58 ± 2.02	4.50 ± 2.43	n.s.
Mozzarella springiness	6.33 ± 2.67	6.67 ± 1.50	4.83 ± 2.76	n.s.
Mozzarella chewability	6.50 ± 1.57	5.58 ± 1.83	5.67 ± 1.56	n.s.
Mozzarella/tomato sauce balance	5.08 ± 1.98	4.58 ± 2.43	6.25 ± 2.01	n.s.

Different letters in the same row indicate significant differences. * Significance at *p* < 0.05. n.s. not significant.

## Data Availability

Data is contained within the article.
